# Supplementary figures and images for: Time-course of host cell transcription during the HTLV-1 transcriptional burst
Source: PLoS Pathog. 2022 May 16;18(5):e1010387. doi: 10.1371/journal.ppat.1010387 (PMC9135347; doi:10.1371/journal.ppat.1010387)

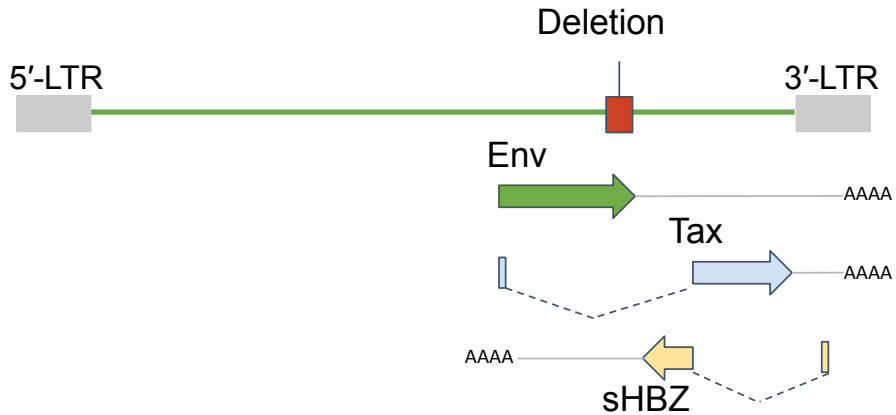

Supplement: S1 Fig — Schematic of the provirus in clone 3.60 with the 202 bp deletion (GenBank: AB513134; coordinates 6420–6621) and coding-regions of Env, Tax and sHBZ marked. (PDF) [file ppat.1010387.s001.pdf]

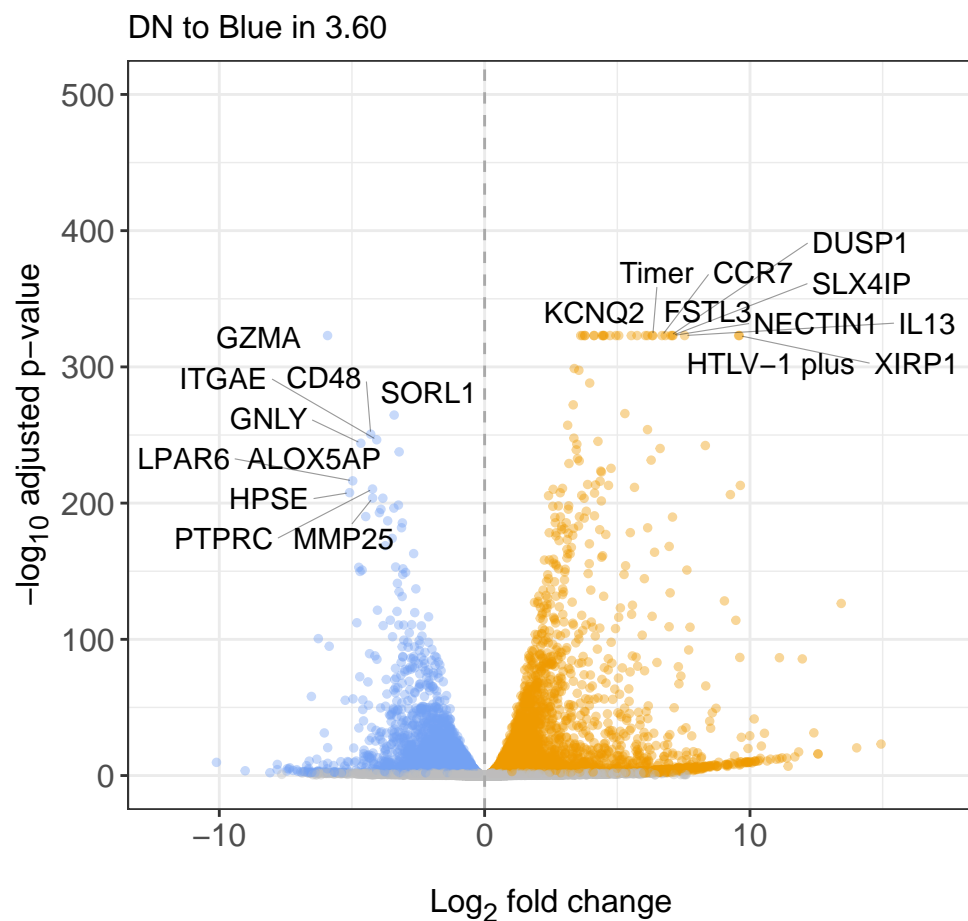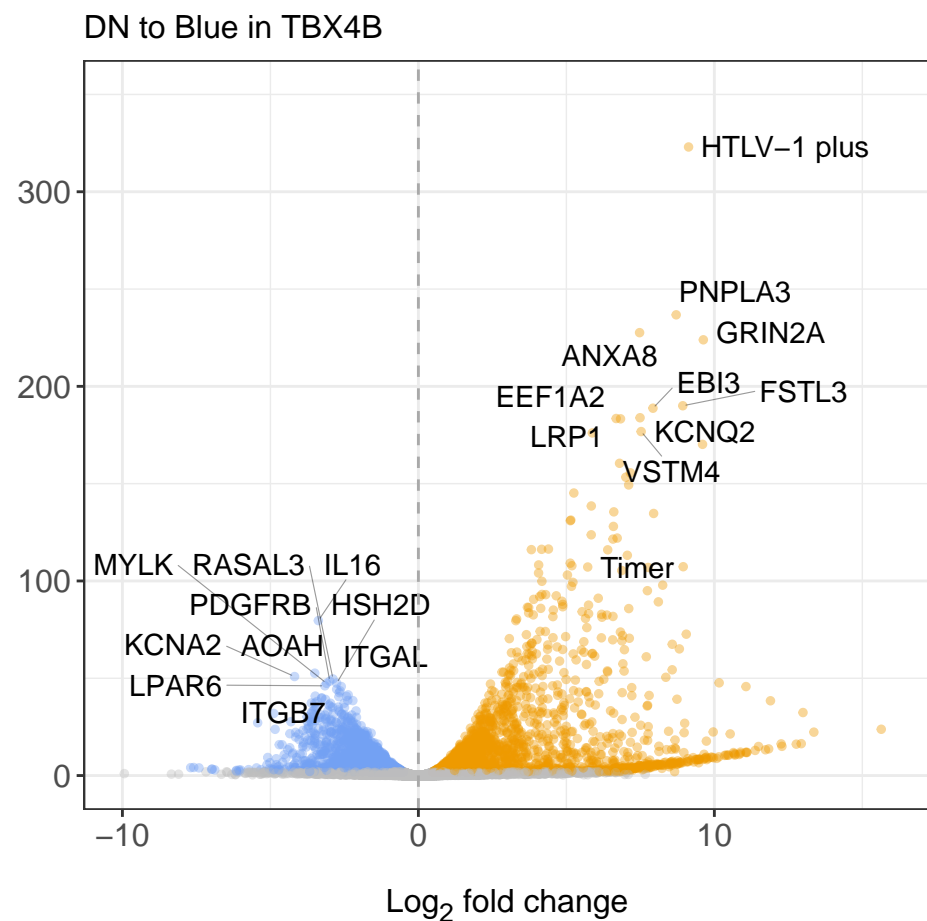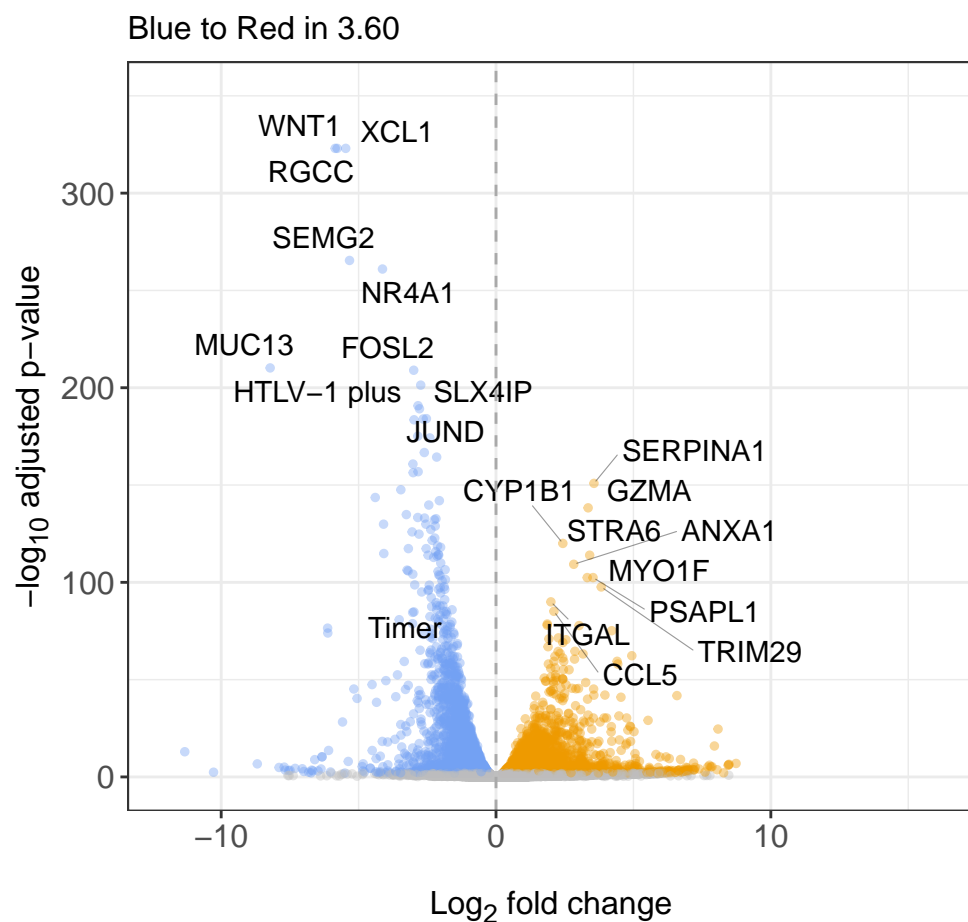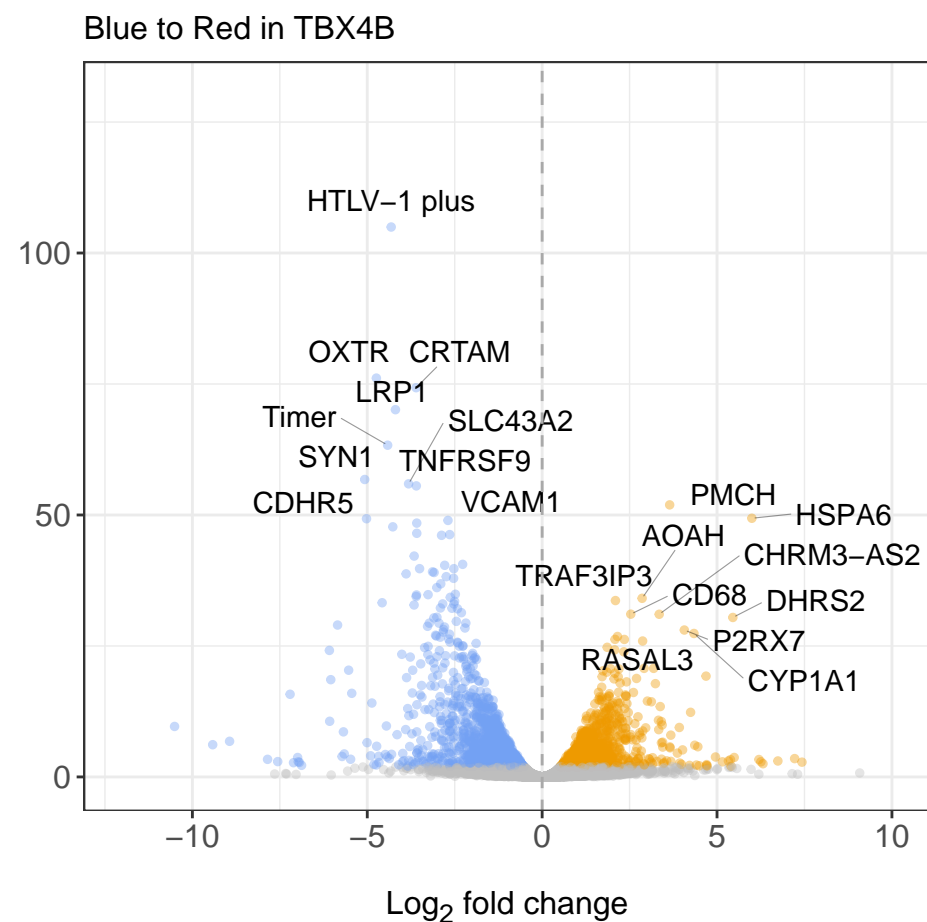

Supplement: S2 Fig — Significantly up-regulated genes are in yellow and down-regulated genes in blue. HTLV-1 plus, Timer and top 10 most significantly up- and down-regulated genes are labelled; ns—not significant. (PDF) [file ppat.1010387.s002.pdf]

A

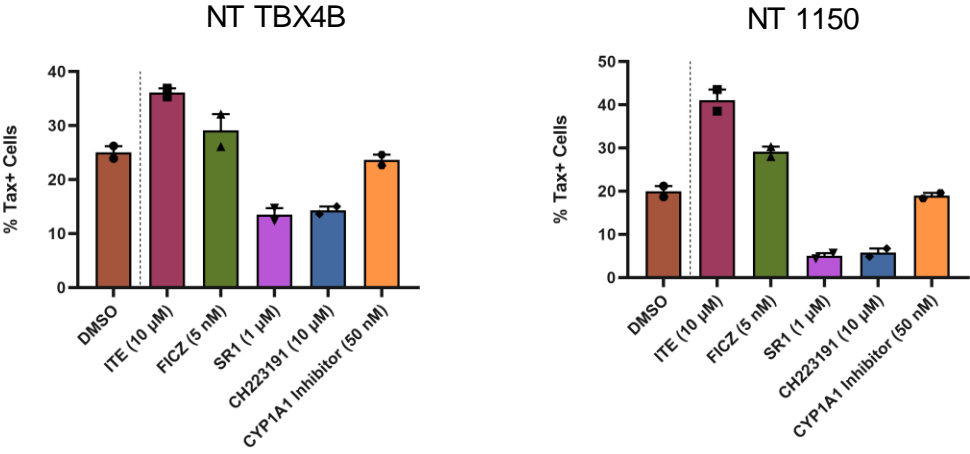

B

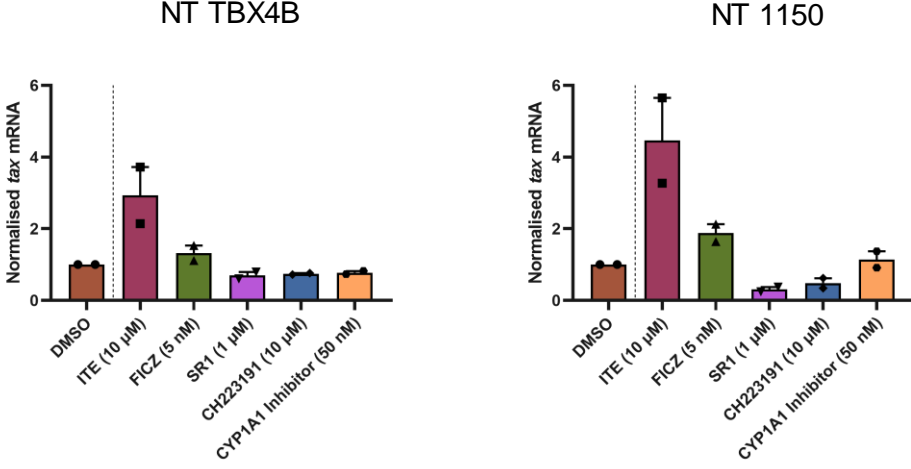

C

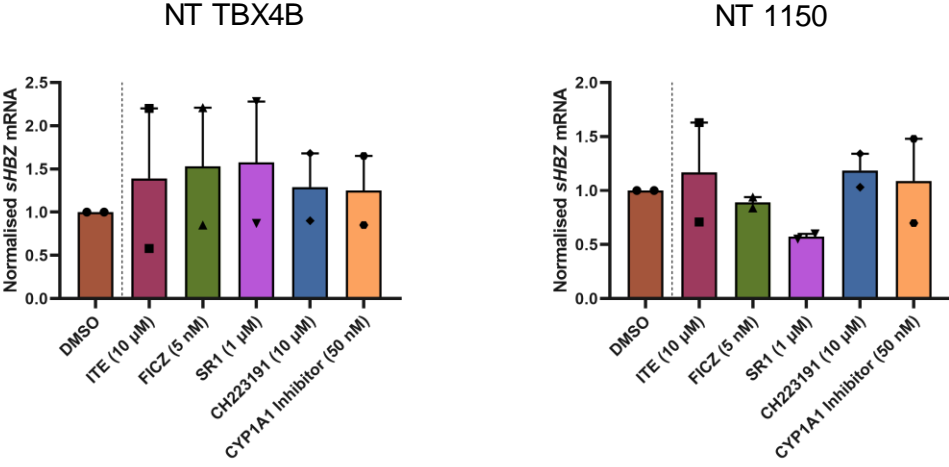

D

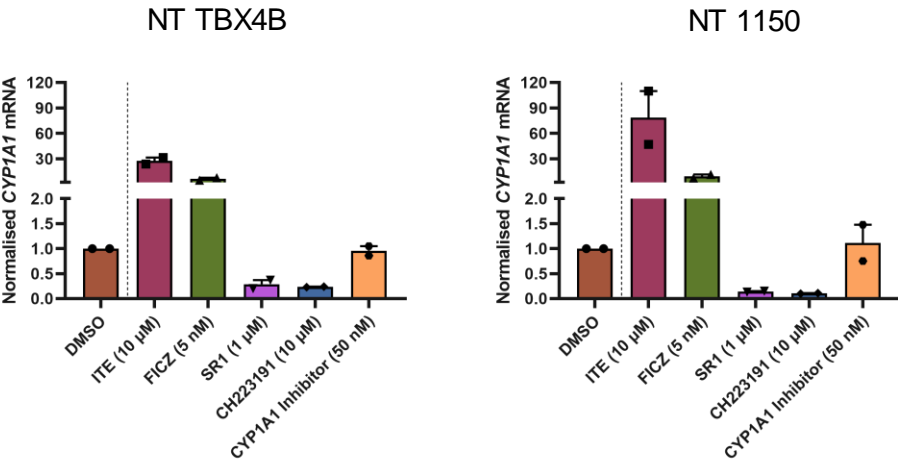

E

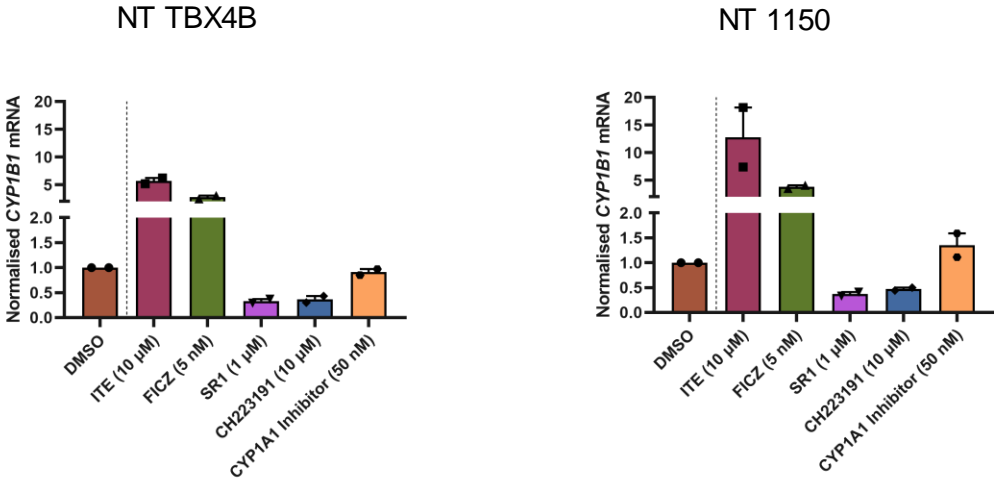

Supplement: S5 Fig — (A) Tax expression analysis by flow cytometry. NT–non-transduced. (B) tax expression analysis RT-qPCR. (C) sHBZ expression analysis RT-qPCR. (D) CYP1A1 expression analysis RT-qPCR. (E) CYP1B1 expression analysis RT-qPCR. (PDF) [file ppat.1010387.s005.pdf]
