# Supplementary material for: Time-course of host cell transcription during the HTLV-1 transcriptional burst
Source: PLoS Pathog. 2022 May 16;18(5):e1010387. doi: 10.1371/journal.ppat.1010387 (PMC9135347; doi:10.1371/journal.ppat.1010387)
Supplement: S3 Fig — Y-axis: normalized counts on log10-scale. Significance is determined with LRT. FDR-corrected p-value < 0.01. (PDF) [file ppat.1010387.s003.pdf]

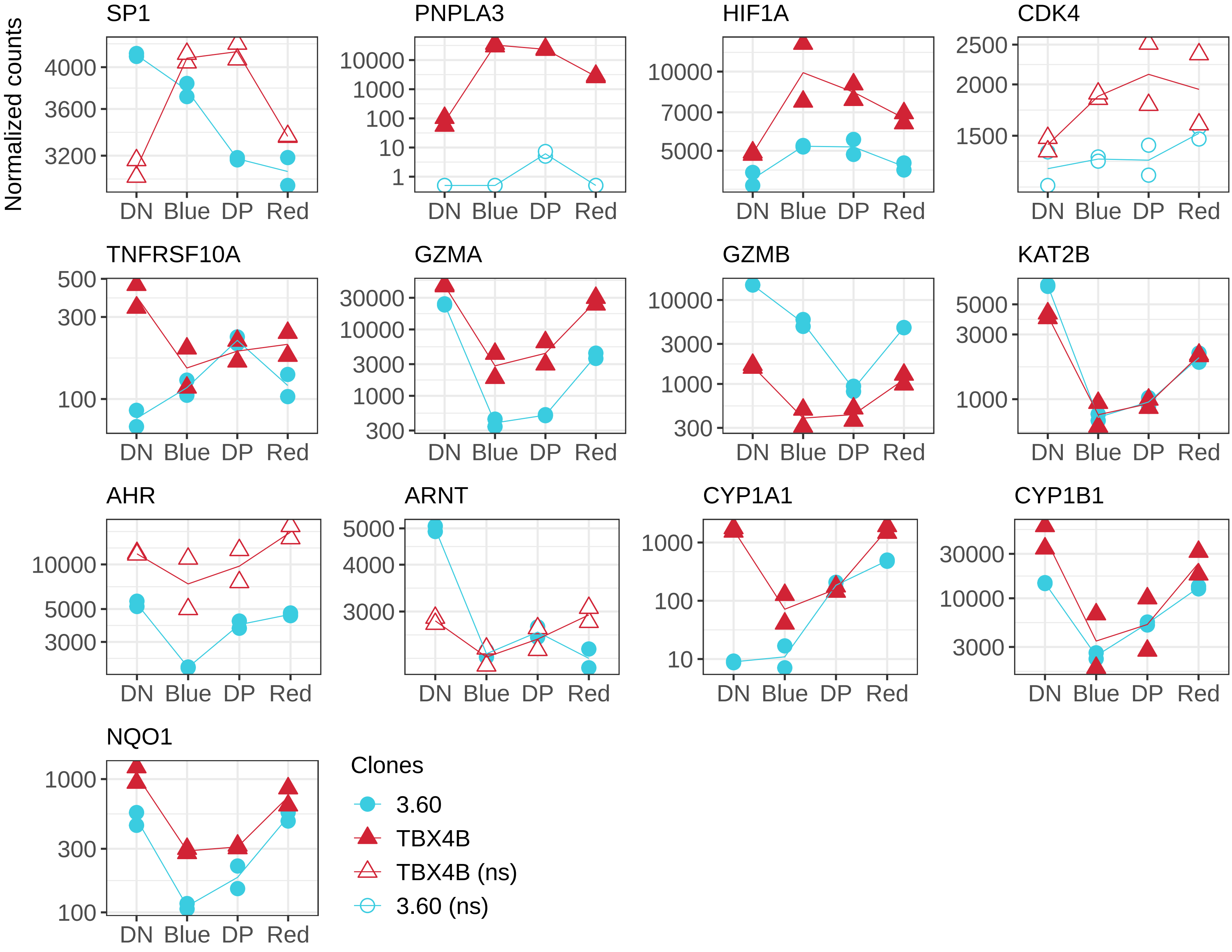
Normalized counts
SP1
PNPLA3
HIF1A
CDK4
TNFRSF10A
GZMA
GZMB
KAT2B
AHR
ARNT
CYP1A1
CYP1B1
NQO1
DN
Blue
DP
Red
Clones
3.60
TBX4B
TBX4B (ns)
3.60 (ns)
