# Supplementary material for: Time-course of host cell transcription during the HTLV-1 transcriptional burst
Source: PLoS Pathog. 2022 May 16;18(5):e1010387. doi: 10.1371/journal.ppat.1010387 (PMC9135347; doi:10.1371/journal.ppat.1010387)
Supplement: S4 Fig — (A) K-means clustering of 10048 significantly DE genes with k = 2 in clone 3.60 and 4798 DE genes in clone TBX4B. The top 10 genes based on mean rank of sorted p-values are listed on the right in each panel. The mean expression trajectory is coloured yellow or blue. Significance is determined with LRT. FDR-corrected p-value < 0.01. (B) Over-representation analysis of K-means clusters with the Hallmarks gene set from The Molecular Signatures Database (MSigDB). Statistical significance was determined by Fisher’s exact test in g:Profiler. FDR-corrected p-value < 0.05. (PDF) [file ppat.1010387.s004.pdf]

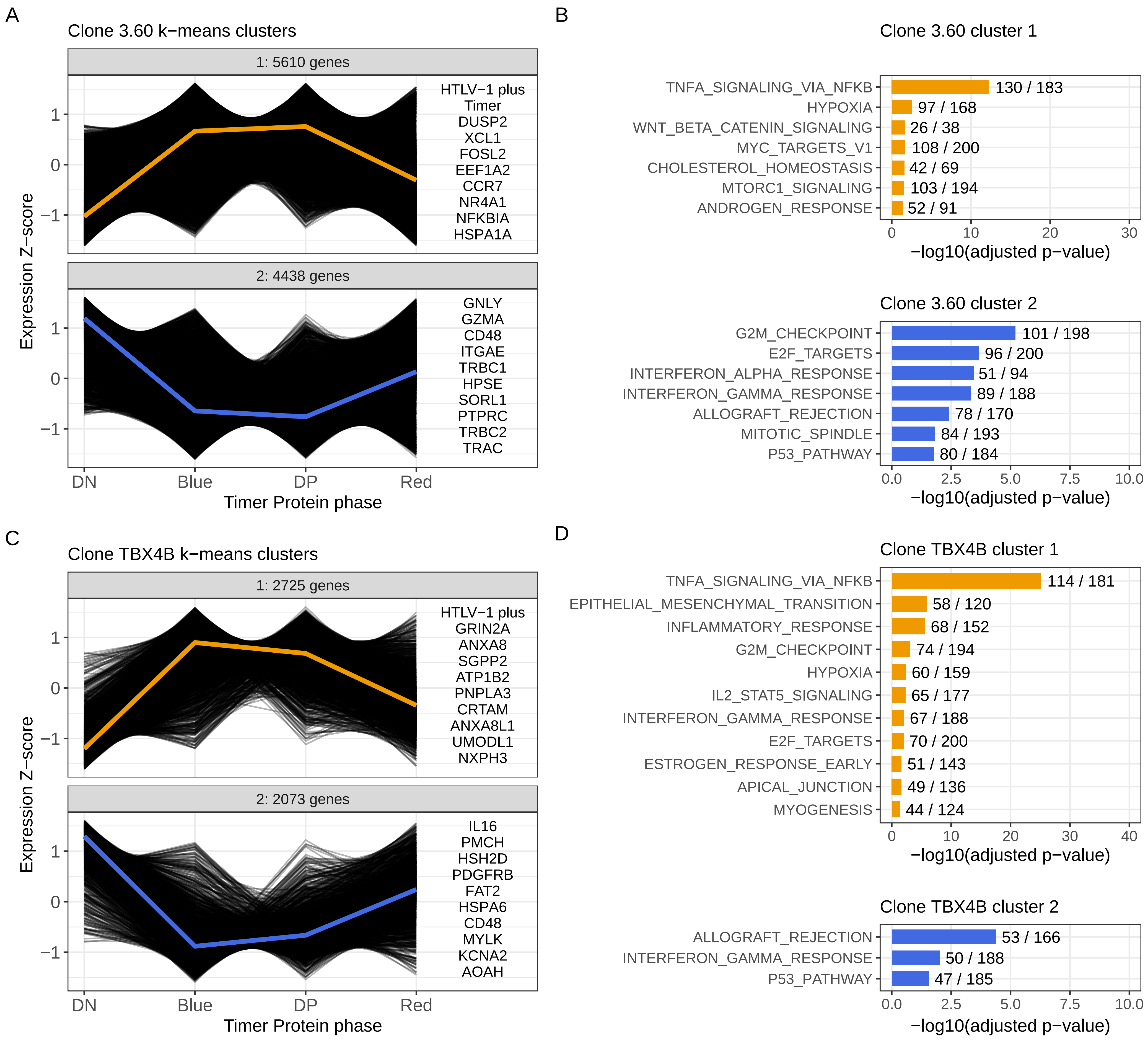
A
Clone 3.60 k−means clusters
1: 5610 genes
HTLV−1 plus
Timer
DUSP2
XCL1
FOSL2
EEF1A2
CCR7
NR4A1
NFKBIA
HSPA1A
2: 4438 genes
GNLY
GZMA
CD48
ITGAE
TRBC1
HPSE
SORL1
PTPRC
TRBC2
TRAC
Expression Z−score
DN
Blue
DP
Red
Timer Protein phase
B
Clone 3.60 cluster 1
TNFA_SIGNALING_VIA_NFKB 130 / 183
HYPOXIA 97 / 168
WNT_BETA_CATENIN_SIGNALING 26 / 38
MYC_TARGETS_V1 108 / 200
CHOLESTEROL_HOMEOSTASIS 42 / 69
MTORC1_SIGNALING 103 / 194
ANDROGEN_RESPONSE 52 / 91
−log10(adjusted p−value)
Clone 3.60 cluster 2
G2M_CHECKPOINT 101 / 198
E2F_TARGETS 96 / 200
INTERFERON_ALPHA_RESPONSE 51 / 94
INTERFERON_GAMMA_RESPONSE 89 / 188
ALLOGRAFT_REJECTION 78 / 170
MITOTIC_SPINDLE 84 / 193
P53_PATHWAY 80 / 184
−log10(adjusted p−value)
C
Clone TBX4B k−means clusters
1: 2725 genes
HTLV−1 plus
GRIN2A
ANXA8
SGPP2
ATP1B2
PNPLA3
CRTAM
ANXA8L1
UMODL1
NXPH3
2: 2073 genes
IL16
PMCH
HSH2D
PDGFRB
FAT2
HSPA6
CD48
MYLK
KCNA2
AOAH
Expression Z−score
DN
Blue
DP
Red
Timer Protein phase
D
Clone TBX4B cluster 1
TNFA_SIGNALING_VIA_NFKB 114 / 181
EPITHELIAL_MESENCHYMAL_TRANSITION 58 / 120
INFLAMMATORY_RESPONSE 68 / 152
G2M_CHECKPOINT 74 / 194
HYPOXIA 60 / 159
IL2_STAT5_SIGNALING 65 / 177
INTERFERON_GAMMA_RESPONSE 67 / 188
E2F_TARGETS 70 / 200
ESTROGEN_RESPONSE_EARLY 51 / 143
APICAL_JUNCTION 49 / 136
MYOGENESIS 44 / 124
−log10(adjusted p−value)
Clone TBX4B cluster 2
ALLOGRAFT_REJECTION 53 / 166
INTERFERON_GAMMA_RESPONSE 50 / 188
P53_PATHWAY 47 / 185
−log10(adjusted p−value)
