# Supplementary material for: Time-course of host cell transcription during the HTLV-1 transcriptional burst
Source: PLoS Pathog. 2022 May 16;18(5):e1010387. doi: 10.1371/journal.ppat.1010387 (PMC9135347; doi:10.1371/journal.ppat.1010387)
Supplement: Attachment — Submitted filename: Response_to_reviewers_Timerprotein.docx [file ppat.1010387.s009.docx]

We thank the reviewers for their careful reading of the manuscript and their helpful comments. Our responses are listed below.

Major Issues

Reviewer #1:

1) Thank you for the comment. The aim of this study was to identify the major changes in host-cell gene expression that accompany the successive phases of the plus-strand proviral burst of HTLV-1 transcription. At present it is impractical to carry out these high-throughput techniques (transduction with the Timer protein followed by RNA-seq analysis) on a large number of clones (and each infected host carries many thousands of infected T-cell clones). Rather, we provide here proof of principle of certain key cellular transcriptional responses in naturally HTLV-1-infected T-cell clones undergoing spontaneous proviral expression.

2) We agree that it will be important to test the hypotheses raised by the present analysis by examining the consequences of the transcriptional changes observed. Indeed in a subsequent study (manuscript in preparation) we have carried out experiments following the trajectory of apoptosis, DNA damage, cellular proliferation, cell motility, chemotaxis, cell clumping, and the dynamics of proviral protein expression in naturally-infected T cell clones. However, it is beyond the scope of the present paper to include these extensive results in addition to the RNA-seq analysis.

Reviewer #2:

1) The reviewer is of course correct that some of the observed responses may be indirect or mediated by other products of the virus. We have clarified this important point in the updated text on lines 409-411. In this work we used Tax protein expression as a marker of plus-strand expression; in this design it is not possible to distinguish the downstream effects of other proviral proteins. Furthermore, in short-read RNA-seq it is difficult to confidently measure the abundance of individual proviral transcripts, which are generated from long proviral transcripts by alternative splicing, and often share part of their sequence. To answer this question precisely it would be important to follow the time-course of production of each viral protein, which at present is impractical. The amended section now reads:

“Some of these observed effects may be due to other plus-strand encoded products; however, the cells were sorted on the expression of the Timer protein, which is directly induced by Tax.“

Minor issues

Reviewer #1:

1) The proviral integration site in each clone has been added to Table 1 in the Materials and Methods.

2) We thank the reviewer for this very interesting question, which is one we have taken particular interest in. We are indeed currently investigating, using live-cell imaging and mathematical modelling, the burst heterogeneity (e.g. interval length and duration) of several clones, which will be part of the functional study described above.

Reviewer #2:

1. Thank you for pointing this out. We have amended Fig. S3 to include CDK4, which is mentioned in line 223. CDK2 is currently present on Fig. 4B.

2. TNFRSF10A has been added to S3 Fig and referenced accordingly within text.

3. The experimental procedure used to generate Figures 8 F and G is explained in detail in "Live cell imaging" in Materials and Methods. The aim here was to study the impact of the AhR pathway on cells already expressing Tax protein, in comparison with non-Tax-expressing cells. We used cells transduced with a fluorescent (d2EGFP) Tax-responsive reporter construct. In each experiment, we flow-sorted at least 6000 cells from the d2EGFP-transduced clone TBW 11.50, based on Tax (d2EGFP) expression. Using the d2EGFP reporter, the cells can be ‘synchronized’ for Tax expression, but cannot be synchronized more precisely – as with the timer protein – for successive phases of the plus-strand burst.

4. We have added the individual trajectories of the two clones used in supporting information as S5 Fig. As seen in the plots representing individual clones, both clones displayed a similar pattern of plus-strand expression at the mRNA and protein levels in response to AhR agonists and antagonists. As mentioned above in response to reviewer #1, we are testing proof of principle concepts; each patient has more than 10 000 clones, requiring a different approach to test population-average effects.

5. We thank the reviewer for raising this point. For the purpose of the initial submission we used the Preflight Analysis and Conversion Engine (PACE) digital diagnostic tool as requested by PLoS Pathogens, which compresses the image and seems to have degraded the quality of our high-resolution images.

Reviewer #3: Below are my comments/suggestions to improve the quality of this paper.

1. Thank you for asking us to modify and clarify this point. We have amended the text on lines 65-67 to read as follows:

“In addition to the canonical retroviral genes, the provirus expresses several regulatory proteins. The sense and antisense strands of the provirus encode the viral transactivator Tax protein and HBZ (HTLV-1 bZIP protein), respectively: these two proteins promote the proliferation and survival of HTLV-1-infected cells, and both have been implicated in the development of ATL [3,4]; however other regulatory proteins may also be involved.”

2. We too were surprised to see KAT2B (P/CAF) transcriptionally downregulated in both clones, which indicates this is a clone-independent effect, and suggests that KAT2B up-regulation does not play a significant part in proviral expression. Further work is needed to understand the role of KAT2B in HTLV-1 plus-strand expression.

3. The filters applied to the data included the removal of non-expressing genes (genes were included for analysis if detected at a minimum of 3 reads in 2 or more of the samples); the default independent filtering performed by DESeq2; and an FDR adjusted p-value < 0.01. We did not apply a general fold-change filter in addition, because of the unclear functional significance of a given fold change, especially for transcription factors and enzymes. In the majority of analyses described, we focus on genes whose expression was significantly changed in both clones, which further suggests the biological relevance of these changes.

4. Indeed, while this has not been formally quantified, on lines 446-447 we note that clone 3.60 also grows more slowly in cell culture. The growth properties and heterogeneity of naturally infected T-cell clones are studied in more detail in the functional study mentioned (see response to reviewer 1).

5. Thank you for this comment. We agree that this observation identifies one of several concepts that remain to be fully explained, including NER as well as KAT2B regulation discussed above, and their association with HTLV-1 expression.
